# Persistent environmental endocrine-disrupting chemicals in ovarian follicular fluid and *in vitro* fertilization treatment outcome in women

**DOI:** 10.1080/03009734.2020.1727073

**Published:** 2020-02-25

**Authors:** Richelle D. Björvang, Pauliina Damdimopoulou

**Affiliations:** Division of Obstetrics and Gynecology, Department of Clinical Science, Intervention and Technology, Karolinska Institutet and Karolinska University Hospital, Stockholm, Sweden

**Keywords:** Assisted reproduction, endocrine disrupting chemical, follicular fluid, persistent organic pollutant

## Abstract

Several international organizations have recently highlighted endocrine-disrupting chemicals (EDCs) as factors of concern in human reproduction. Since successful reproduction is dependent on timely and appropriate action of hormones, disruption of the endocrine system could lead to difficulties in conceiving or carrying a pregnancy to term. EDCs are chemicals that disrupt the endocrine system by activating or inhibiting receptors of the endocrine system, and/or altering hormone receptor expression; signal transduction; epigenetic marks; hormone synthesis, transport, distribution, and metabolism; and the fate of hormone-producing cells. Due to the increasing production of industrial chemicals over the past century and their lenient control, EDCs are now common contaminants in the environment. Consequently, everyone faces a life-long exposure to mixtures of chemicals, some of which have been identified as EDCs. As birth rates in humans are declining and the use of assisted reproductive technologies increasing, it is timely to consider possible effects of EDCs on human reproduction and fertility. In this review, we focus on persistent EDCs, their occurrence in ovarian follicular fluid, and associations to treatment outcomes in assisted reproduction. Our summary shows that despite being banned decades ago, mixtures of persistent EDCs are still detected in the ovarian follicular fluid, demonstrating direct exposure of oocytes to these chemicals. In addition, there are several reported associations between exposure and worse outcome in *in vitro* fertilization. Further research is therefore warranted to prove causality, which will lead towards better regulation and exposure reduction.

## 1. Introduction

The phenomenon of endocrine disruption started gaining attention in the 1990s after a group of experts concluded that many compounds introduced into the environment by human activity are capable of disrupting the endocrine system of animals and humans with possibly profound consequences (1). The concept of endocrine disruption was popularized by Theo Colborn’s book, *Our Stolen Future*, that proposed that chemical pollution is threatening the intelligence, fertility, and survival of the human race (2). Today, 25 years after the term endocrine-disrupting chemical (EDC) was coined, endocrine disruption remains a highly relevant area of research and debate in society, and the methods to identify and regulate these chemicals are still under development.

There are no international registries of numbers of chemicals in the market. The US Toxic Substance Control Act (TSCA) inventory contains over 86,000 existing chemicals. In the European Union, over 22,000 unique substances are registered under the Registration, Evaluation, Authorisation and Control (REACH) regulation. These databases only have chemicals produced or imported over 10,000 kg/year (TSCA) or 1000 kg/year (REACH), so it is safe to assume the actual number of different chemicals that are or have been in the market is higher^1^. The chemical industry is one of the most profitable businesses in the world with a revenue of US$5.7 trillion in 2019. The biggest chemical producers being China, Europe, and the United States (4,5).

Unfortunately, the speed of production of new chemicals has far exceeded the speed of development of chemical health risk assessment. The side effects of uncontrolled chemical use were first discovered in 1950s when wildlife populations of birds, reptiles and mammals started drastically declining due to uncontrolled use of organochlorine pesticides like DDT (dichlorodiphenyltrichloroethane), lindane (*gamma*-hexachlorocyclohexane), and chlordane (octachloro-4,7-methanohydroindane). In the Baltic Sea region, organochlorine chemicals nearly caused the extinction of the Baltic grey seal and the white-tailed sea eagle (6,7). In the United States, populations of bald eagles and alligators declined in polluted areas (8,9). These alarming occurrences among others led little by little to the establishment of international agreements for the restriction of chemicals, such as the Stockholm Convention (ratified in 2004) as well as to the development of tests for chemical risk assessment. By the time the first validated OECD guidelines for the testing of chemicals were in place in the 1980s, thousands of chemicals were already in the market.

Although organochlorine chemicals were regulated starting from the 1970s, and later internationally restricted by the Stockholm Convention, they still persist in the environment due to their extremely long half-lives. Sadly, they also still threaten the reproductive success and survival of long-lived species like killer whales (10). In addition, they are now accompanied by a plethora of newer chemicals. Current requirements for chemical safety testing in the European Union (and elsewhere) are imperfect, in particular for endocrine-disruptive activity (11). The required regulatory structure having a clear definition of EDCs, guidance documents, suitable tests, test requirements, and risk management is not in place for any sector of chemical legislation (11). In practice, this means that no regulatory risk assessment concerning endocrine-disruptive activity has been carried out for the chemicals currently in the market. According to estimates by the United Nations Environment Programme and World Health Organization (UNEP/WHO), there are at least 800 chemicals with known endocrine-disruptive activity (12). The European Union has formally recognised 13 chemicals as EDCs (11).

Several international organisations in the field of public and reproductive health have recently expressed their concerns about EDCs and human reproduction. UNEP/WHO prepared an extensive summary of EDCs in 2012 and concluded that there are many gaps in our knowledge of endocrine disruption of the female reproductive system and that test methods for screening of chemicals for endocrine disruption on female reproduction are missing (12). A few years later, the Endocrine Society released their second scientific summary on EDCs stating that several classes of chemicals ranging from pesticides to plasticisers can impair ovarian development and function, suggesting that exposure to EDCs may be associated, for example, with reduced fertility, infertility, polycystic ovarian syndrome, endometriosis, and fibroids (13). Following this, Trasande and colleagues estimated that the uncontrolled use of EDCs in Europe is associated with increased incidence of uterine fibroids and endometriosis, with an estimated annual cost of 1.4 billion euros to the taxpayers (14). In 2013, the American College of Obstetricians and Gynaecologists (ACOG) published a committee opinion on exposure to toxic environmental agents stating that ‘the evidence that links exposure to toxic environmental agents and adverse reproductive and developmental health outcomes is sufficiently robust’ to call for timely action to identify and reduce exposure while addressing the consequences (15). The International Federation of Gynaecology and Obstetrics (FIGO) joined this view in their 2015 opinion (16). Both ACOG and FIGO also acknowledge that while the exposure to chemicals is ubiquitous, it disproportionally affects people with low income. Hence, actions taken to prevent harm of EDC exposure in women is not only a question of gender equality, but also a matter of equality in society at large.

With this review, we wish to bring the attention of the clinicians working with reproductive-age patients to environmental chemicals as factors affecting fertility and reproductive health in women. We will first briefly outline some central concepts of endocrine disruption, and then focus on three topics: the extensive mixture exposure of all populations to industrial chemicals; the occurrence of persistent environmental chemicals with endocrine-disruptive activities in patients seeking assisted reproduction; and the potential implications of this exposure.

## 2. Central concepts of endocrine disruption

### 2.1. Definitions and mechanism of action

An EDC is defined as an ‘exogenous substance or mixture that alters function(s) of the endocrine system and consequently causes adverse health effects in an intact organism, or its progeny, or (sub)populations’ (17). This definition is complex as it needs both an endocrine activity and a demonstration of adverse effects as its consequence in living organisms. Adversity in the context of endocrine disruption is defined as ‘a change in morphology, physiology, growth, reproduction, development or lifespan of an organism which results in impairment of functional capacity or impairment of capacity to compensate for additional stress or increased susceptibility to the harmful effects of other environmental influences’ (18).

There is a wide range of mechanisms by which EDCs can interfere with the endocrine system and cause adverse effects. Classically, EDCs are thought to act via receptor-mediated disruption where they mimic actions of endogenous hormones such as oestrogen and androgen (agonists) or blocking interaction of the ligand with the receptor (antagonists) (19). Recently, ten key characteristics for the identification of EDCs have been proposed (19). In addition to activation or inhibition of receptors of the endocrine system, alteration of hormone receptor expression, signal transduction, epigenetic marks, hormone synthesis, transport, distribution and metabolism, and/or the fate of hormone-producing cells are listed as mechanisms of action of EDCs (19).

In the classical mechanism of action, EDCs bind to nuclear hormone receptors, which then bind to specific response elements and influence transcription of their target genes (18). By contrast, they can also act as antagonists by binding to the receptor but not triggering the normal response (18). For example, oestrogen-disruptive activity could result from the EDC binding to the oestrogen receptor and subsequently activating (agonist) or repressing (antagonist) its downstream activity in the cell. In addition, many EDCs also bind to the aryl hydrocarbon receptor (AhR), which like the hormone receptors is a ligand-activated transcription factor. AhR is evolutionary conserved, widely expressed, and activated by a variety of xenobiotics. In response, it triggers the expression of genes involved in xenobiotic and hormone metabolism, such as CYP enzymes (e.g. *CYP1A1*) and *UDPGT1A*, by binding to specific response elements on DNA in the promoters of these genes (18). Moreover, AhR can also cross-talk with other nuclear receptors, implying that it can indirectly interfere with hormonal signalling pathways at large (18).

### 2.2. Features of endocrine disrupting chemicals

EDCs have diversified the field of toxicology by challenging traditional toxicological dogmas. It was originally thought that substances cause toxicity in a monotonic dose–response with consequences seen at high doses, that is, dose makes the poison. However, this is not the case with EDCs. Similar to natural hormones, EDCs can produce non-monotonic dose–response curves, where the slope of the curve changes from positive to negative or vice versa, thereby having a U- or inverted U-shape. Some of the mechanisms behind this response are receptor selectivity, receptor competition, feedback loops, and receptor number (20). This can lead to significant effects even at low doses, implying that biological effects can be observed at exposure levels typical to human exposure or lower. The endocrine system responds to very low concentrations of endogenous hormones due to high affinity of hormones to their receptors, among others. Similarly, as EDCs mimic natural hormones, they can also trigger a response at low levels (21). For example, the plastic additive bisphenol A leaching from plastic mouse cages caused disruption of meiotic spindles in mouse oocytes at exposure levels corresponding to 1 μg/day per mouse (22). For comparison, the estimated human intake of bisphenol A varies between 10–60 ng/kg per day, suggesting exposure of 0.6–3.6 μg/day for a person weighing 60 kg (23).

It has also been shown that there can be a long lag time from exposure until the adverse effect is seen. For example, exposure to EDCs during organogenesis is associated with increased risk of development of diseases later in life (24). Moreover, this also suggests that chemicals can cause more damage when exposure takes place during certain windows of susceptibility such as the prenatal and early postnatal period because they disrupt essential organ development (25,26). An example of this is diethylstilbestrol (DES), a synthetic non-steroidal oestrogen prescribed from 1930s to 1970s to prevent miscarriages as well as decrease risk of pregnancy complications and premature delivery. As DES interfered with the reproductive tract development *in utero*, DES-exposed daughters had higher primary infertility, were less likely to have full-term births, and had higher likelihood of premature births, spontaneous miscarriages, and ectopic pregnancies compared with unexposed women (27–29). The DES incidence has also illustrated the multigenerational effects of EDCs as the grandchildren of DES-exposed women have increased risk of irregular menstrual cycles, amenorrhoea, ectopic pregnancy, and preterm delivery (30).

Since EDCs are ubiquitous and can be found in various consumer products, we are not exposed to a single chemical but to multiple chemicals at the same time. Common routes of exposure to EDCs are oral, respiratory, and dermal. They can also enter the body through intravenous, intramuscular, or subcutaneous routes for example during medical treatments such as IVF procedures. Developing foetuses can be exposed through placental transfer of chemicals from the mother, and neonates via breastmilk (12,31). The extensive exposure to EDCs can be seen in various biomonitoring programmes in different countries where pesticides, phthalates, bisphenols, aromatic hydrocarbons, benzophenones, perfluoroalkyl substances (PFAS), chlorinated chemicals, and metals are commonly detected in the general population (32,33). This mixture exposure can lead to combinatory effects of chemicals called cocktail effects. As chemicals are usually assessed individually, the hazards and risks could be underestimated because possible additive (1 + 1 = 2), synergistic (1 + 1 > 2), or antagonistic (1 + 1 < 2) properties are not accounted for. There is continuous effort on designing and optimising statistical approaches to quantifying the effect of mixtures. Various statistical approaches have been proposed from machine learning to classical linear regression, but there is no single best approach that outperforms the others (34).

## 3. Exposures and outcomes in IVF patients

### 3.1. Persistent organic pollutants

There are various different groups of EDCs, but for the purpose of this review we focus on persistent organic pollutants (POPs). POPs are halogenated organic chemical substances that are toxic to both human and wildlife, bioaccumulative, and resistant to environmental degradation because of their stability. While most POPs are lipophilic in nature and accumulate to fatty tissues, PFAS are amphiphilic and bind to proteins. In general, POPs are also volatile at certain temperatures and may travel long distances in the atmosphere. Hence, they can be found even in areas where they were never used (12,35). For humans, the largest source of POP exposure is diet. Contaminated Baltic Sea fish remains a significant source of POPs in Scandinavian countries. A list of the POPs in focus of this review, their uses, and regulations are given in Table 1.

**Table 1.** Use, source, and regulation of POPs and their suggested reproductive health effects in women.

Chemical	Use/sources	Regulation^a^	Associated health effects in women	References
PeCB and HCB	Fungicide; unintentional production during industrial processes	Annex A and C	Failed implantation, increased spontaneous abortion	Mahalingaiah et al. (36); Younglai et al. (37)
HCH (lindane)	Agricultural insecticide and treatment for lice and scabies	Annex A	Increased spontaneous abortion, premature delivery, endometriosis	Upson et al. (38); US Department of Health and Human Services (39)
Chlordane	Termite treatment in food crops (e.g. corn and citrus)	Annex A	Altered cycle length	Chen et al. (40)
DDT and DDE	Disease vector control (e.g. malaria)	Annex B	Impaired fertilization, impaired lactation, infertility, reduced parity, longer time-to-pregnancy, uterine fibroids	Gesink Law et al. (41); Trabert et al. (42); Younglai et al. (37) (43)
PCBs	Electrical insulation, heat transfers, hydraulic systems and capacitors, paints, plasticizers, dyes for carbonless duplicating paper	Annex A and C	Impaired response to ovulation induction, impaired lactation, reduced parity and fecundability, longer time to pregnancy, uterine fibroids	Gennings et al. (44); Gesink Law et al. (41); Trabert et al. (42); Younglai et al. (37)
PBDEs	Flame retardants added to fabrics, textiles, plastics, carpets, and electronical appliances	Annex A	Failed implantation, decreased fecundability, endometriosis	Johnson et al. (45); Harley et al. (46); Ploteau et al. (47)
PFASs	Consumer products that are water-, oil-, and stain-resistant (e.g. Scotchgard, Teflon)	Annex B	Longer time-to-pregnancy, infertility, endometriosis	Buck Louis et al. (48); Campbell et al. (49); Fei et al. (50)

^a^Regulation under the Stockholm Convention: Annex A, elimination of production and use; Annex B, restrict production and use; Annex C, reduce unintentional releases.

DDE: dichlorodiphenyldichloroethylene; DDT: dichlorodiphenyltrichloroethane; HCB:hexachlorobenzene; HCH: hexachlorocyclohexane; PBDE: polybrominated diphenyl ether; PCB: polychlorinated biphenyl; PeCB: pentachlorobenzene; PFAS: perfluoroalkyl substance.

We choose to focus on POPs since organochlorine chemicals, which form a large part of the group, are historically the chemicals that are associated with disruption of reproductive activities in wildlife. They accumulate in humans with increasing age due to their long half-lives, and the levels therefore reflect the life-history of exposure. Currently, women postpone childbearing. The average age of first-time mothers in Sweden is 27.3 years for the whole country, and 30.3 years for its capital Stockholm (51). Delaying starting of a family means longer cumulative exposure to environmental factors, including POPs. Particularly for older women, whose oocyte quality is already declining (52), the increasing cumulative exposure to chemicals could further worsen the chances of pregnancy. In contrast to other EDCs that are easily metabolised such as phthalates, it is difficult to reduce the body burden of POPs only through lifestyle modification. For women, the body burden of lipophilic POPs is reduced when bearing a child because these chemicals cross the placenta and deposit to the foetus (53). In addition, they are also transferred to the neonate via breast milk (54).

### 3.2. POPs in follicular fluid and associations to outcomes

With the advent of assisted reproductive technologies, follicular fluid has become accessible for evaluating the direct exposure of oocytes to EDCs. Several studies have measured concentrations of POPs in follicular fluid, and some also analysed the associations to treatment outcomes. We summarise the literature on POPs in follicular fluid in Table 2. The lipophilic POPs have been adjusted for sample lipid content in some studies. Although the exposure levels were reported in different units (e.g. ng/mL or ng/g wet weight or ng/g lipids), which made direct comparisons between studies challenging, the summary shows direct exposure of oocytes to mixtures of POPs, which could lead to cocktail effects. Only three studies (57,61,65) gave account of this mixture exposure with the use of principal component analysis.

**Table 2.** Levels of POPs in follicular fluid and the associations to IVF outcome.

Chemical	Sampling period	*n*	Age (years)	Location	Level in follicular fluid: Mean {Geometric mean} [Median] (SD or range)^a^	Association to IVF outcomes^b^	References
HCB	1994–2003	72	25–44	USA	[0.035] ng/g wet weight	–	Meeker et al. (55)
	2000	12	28–32	Italy	73 ng/g lipids	–	De Felip et al. (56)
	2008–2009	40	25–43	Belgium	32 (19) pg/mL	Lower fertilization rate; fewer high-quality embryos	Petro et al. (57)
DDE	1994–2003	72	25–44	USA	[0.363] ng/g wet weight	–	Meeker et al. (55)
	2000	12	28–32	Italy	630 ng/g lipids	–	De Felip et al. (56)
	2002–2003	619	19–50	Saudi Arabia	0.407 μg/L	No association with IVF outcomes	Al-Saleh et al. (58)
	2003–2004	99	25–41	Czech Republic	3303.3 (4205.2) ng/g lipids	No association with IVF outcomes	Jirsová et al. (59)
	2007–2008	32	28–42	USA	0.68 (0.92) ng/mL	Higher peak oestradiol level; lower likelihood for retrieval of mature oocyte	Bloom et al. (60)
	2008–2009	40	25–43	Belgium	392 (348) pg/mL	Lower fertilization rate; fewer high-quality embryos	Petro et al. (57)
	2013	127	20–35	China	84.3 (34.2) ng/g lipids	–	Zhu et al. (61)
	NR	21	28–38	Canada	2677 (1584) pg/mL	Negatively correlated with fertilization	Younglai et al. (43)
DDD	2002–2003	619	19–50	Saudi Arabia	0.0004 μg/L	No association with IVF outcomes	Al-Saleh et al. (58)
	2003–2004	99	25–41	Czech Republic	13.1 (15.6) ng/g lipids	No association with IVF outcomes	Jirsová et al. (59)
	2013	127	20–35	China	5.8 (16.8) ng/g lipids	–	Zhu et al. (61)
DDT	1994–2003	72	25–44	USA	[0.014] ng/g wet weight	–	Meeker et al. (55)
	2002–2003	619	19–50	Saudi Arabia	0.0044 μg/L	No association with IVF outcomes	Al-Saleh et al. (58)
	2003–2004	99	25–41	Czech Republic	98.1 (75.9) ng/g lipids	No association with IVF outcomes	Jirsová et al. (59)
	2007–2008	32	28–42	USA	0.01 (0.03) ng/mL	No association with IVF outcomes	Bloom et al. (60)
	2008–2009	40	25–43	Belgium	35 (5) pg/mL	No association with IVF outcomes	Petro et al. (57)
	2010–2013	94	20–38	Egypt	21.1 (3.8) μg/L	Thinner endometrium, lower number of sacs	Al-Hussaini et al. (63)
	2013	127	20–35	China	8.39 (4.16) ng/g lipids	–	Zhu et al. (61)
a-HCH	2013	127	20–35	China	29.3 (15.1) ng/g lipids	–	Zhu et al. (61)
b-HCH	2008–2009	40	25–43	Belgium	34 (35) pg/mL	Lower fertilization rate; fewer high-quality embryos	Petro et al. (57)
	2013	127	20–35	China	27.3 (26.1) ng/g lipids	–	Zhu et al. (61)
g-HCH	2008–2009	40	25–43	Belgium	34 (1) pg/mL	No association with IVF outcomes	Petro et al. (57)
	2013	127	20–35	China	18.5 (6.74) ng/g lipids	–	Zhu et al. (61)
d-HCH	2013	127	20–35	China	72.7 (49.7) ng/g lipids	–	Zhu et al. (61)
Oxychlordane	1994–2003	72	25–44	USA	[0.012] ng/g wet weight	–	Meeker et al. (55)
	2008–2009	20	25–43	Belgium	ND	No association with IVF outcomes	Petro et al. (57)
Transnonachlor	1994–2003	72	25–44	USA	[0.020] ng/g wet weight	–	Meeker et al. (55)
	2008–2009	20	25–43	Belgium	ND	No association with IVF outcomes	Petro et al. (57)
PCB 28	2007–2008	32	28–42	USA	0.13 (0.03) ng/mL	Lower likelihood for implantation	Bloom et al. (60)
	2010–2013	94	20–38	Egypt	45.5 (9.4) μg/L	Thicker endometrium, higher number of eggs retrieved	Al-Hussaini et al. (62)
	2013	127	20–35	China	25 (39) ng/g lipids	–	Huang et al. (63)
PCB 44	2003–2004	99	25–41	Czech Republic	1.8 (2.5) ng/g lipids	No association with IVF outcomes	Jirsová et al. (59)
	2007–2008	32	28–42	USA	0.09 (0.03) ng/mL	No association with IVF outcomes	Bloom et al. (60)
PCB 47	2003–2004	99	25–41	Czech Republic	13.1 (34.0) ng/g lipids	No association with IVF outcomes	Jirsová et al. (59)
PCB 49	2007–2008	32	28–42	USA	0.07 (0.03) ng/mL	No association with IVF outcomes	Bloom et al. (60)
	NR	21	28–38	Canada	62.4 (6.8) pg/mL	Outcome result not shown	Younglai et al. (43)
PCB 52	2007–2008	32	28–42	USA	0.16 (0.04) ng/mL	No association with IVF outcomes	Bloom et al. (60)
	2010–2013	94	20–38	Egypt	370.6 (54.1) μg/L	Thinner endometrium, higher number of sacs	Al-Hussaini et al. (62)
	2013	127	20–35	China	43 (37) ng/g lipids	–	Huang et al. (63)
PCB 66	2007–2008	32	28–42	USA	0.09 (0.04) ng/mL	Lower likelihood for implantation and live birth	Bloom et al. (60)
PCB 74	2007–2008	32	28–42	USA	0.06 (0.02) ng/mL	Lower likelihood for live birth	Bloom et al. (60)
PCB 87	2007–2008	32	28–42	USA	0.05 (0.02) ng/mL	Thinner endometrium; lower embryo quality	Bloom et al. (60)
PCB 99	2000	12	28–32	Italy	20 ng/g lipids	–	De Felip et al. (56)
	2007–2008	32	28–42	USA	0.04 (0.02) ng/mL	Thinner endometrium	Bloom et al. (60)
PCB 101	2000	12	28–32	Italy	40 ng/g lipids	–	De Felip et al. (56)
	2003–2004	99	25–41	Czech Republic	8.1 (9.1) ng/g lipids	No association with IVF outcomes	Jirsová et al. (59)
	2007–2008	32	28–42	USA	0.11 (0.04) ng/mL	Lower likelihood for live birth	Bloom et al. (60)
	2013	127	20–35	China	ND	–	Huang et al. (63)
PCB 105	2007–2008	32	28–42	USA	0.02 (0.02) ng/mL	Lower likelihood for retrieval of mature oocyte; higher likelihood for oocyte fertilisation	Bloom et al. (60)
	2008–2009	40	25–43	Belgium	ND	No association with IVF outcomes	Petro et al. (57)
PCB 110	2007–2008	32	28–42	USA	0.09 (0.04) ng/mL	No association with IVF outcomes	Bloom et al. (60)
PCB 118	1994–2003	72	25–44	USA	[0.032] ng/g wet weight	–	Meeker et al. (55)
	2000	12	28–32	Italy	77 ng/g lipids	–	De Felip et al. (56)
	2007–2008	32	28–42	USA	0.06 (0.04) ng/mL	No association with IVF outcomes	Bloom et al. (60)
	2008–2009	40	25–43	Belgium	15 (8) pg/mL	Lower fertilization rate; fewer high-quality embryos	Petro et al. (57)
	2013	127	20–35	China	9 (19) ng/g lipids	–	Huang et al. (63)
	NR	8	29–44	Belgium	60 (28−87) pg/g wet weight	–	Pauwels et al. (64)
PCB 138	1994–2003	72	25–44	USA	[0.047] ng/g wet weight	–	Meeker et al. (55)
	2000	12	28–32	Italy	330 ng/g lipids	–	De Felip et al. (56)
	2007–2008	32	28–42	USA	0.05 (0.03) ng/mL	Lower peak oestradiol level	Bloom et al. (60)
	2008–2009	40	25–43	Belgium	49 (32) pg/mL	Lower fertilization rate; fewer high-quality embryos	Petro et al. (57)
	2010–2013	94	20–38	Egypt	146.2 (21.3) μg/L	Thicker endometrium	Al-Hussaini et al. (62)
	2013	127	20–35	China	ND	–	Huang et al. (63)
	NR	8	29–44	Belgium	161 (63−396) pg/g wet weight	–	Pauwels et al. (64)
PCB 146	2007–2008	32	28–42	USA	0 (0.01) ng/mL	Higher likelihood for implantation	Bloom et al. (60)
PCB 149	2007–2008	32	28–42	USA	0.05 (0.03) ng/mL	Thinner endometrium; lower embryo quality	Bloom et al. (60)
PCB 151	2007–2008	32	28–42	USA	0.01 (0.01) ng/mL	Fewer baseline antral follicles, fewer oocytes retrieved, and thinner endometrium	Bloom et al. (60)
PCB 153	2000	12	28–32	Italy	500 ng/g lipids	–	De Felip et al. (56)
	1994–2003	72	25–44	US	[0.072] ng/g wet weight	–	Meeker et al. (55)
	2007–2008	32	28–42	USA	0.07 (0.04) ng/mL	Lower peak oestradiol level	Bloom et al. (60)
	2008–2009	40	25–43	Belgium	72 (44) pg/mL	Lower fertilization rate; fewer high-quality embryos	Petro et al. (57)
	2013	127	20–35	China	0.04 (0.28) ng/g lipids	–	Huang et al. (63)
	NR	8	29–44	Belgium	171 (93−411) pg/g wet weight	–	Pauwels et al. (64)
	NR	21	28−38 years	Canada	73.3 (7.0) pg/mL	Outcome result not shown	Younglai et al. (43)
PCB 156	2000	12	28–32	Italy	30 ng/g lipids	–	De Felip et al. (56)
PCB 157	2000	12	28–32	Italy	6.7 ng/g lipids	–	De Felip et al. (56)
PCB 158	2003–2004	99	25–41	Czech Republic	10.2 (10.9) ng/g lipids	No association with IVF outcomes	Jirsová et al. (59)
PCB 167	2000	12	28–32	Italy	25 ng/g lipids	–	De Felip et al. (56)
PCB 170	2000	12	28–32	Italy	100 ng/g lipids	–	De Felip et al. (56)
	2007–2008	32	28–42	USA	0.01 (0.01) ng/mL	Fewer baseline antral follicles	Bloom et al. (60)
	2008–2009	40	25–43	Belgium	21 (13) pg/mL	Lower fertilization rate; fewer high-quality embryos	Petro et al. (57)
PCB 180	2000	12	28–32	Italy	400 ng/g lipids	–	De Felip et al. (56)
	1994–2003	72	25–44	US	[0.045] ng/g wet weight	–	Meeker et al. (55)
	2007–2008	32	28–42	USA	0.04 (0.02) ng/mL	Fewer baseline antral follicles	Bloom et al. (60)
	2008–2009	40	25–43	Belgium	51 (33) pg/mL	Lower fertilization rate; fewer high-quality embryos	Petro et al. (57)
	2010–2013	94	20–38	Egypt	101.5 (19.2) μg/L	Thinner endometrium, lower number of fertilised oocytes, lower number of cleaved embryos	Al-Hussaini et al. (62)
	2013	127	20–35	China	0.03 (0.31) ng/g lipids	–	Huang et al. (63)
	NR	21	28−38 years	Canada	62.2 (5.2) pg/mL	Outcome result not shown	Younglai et al. (43)
	NR	8	29–44	Belgium	161 (64−372) pg/g wet weight	–	Pauwels et al. (64)
PCB 183	2000	12	28–32	Italy	50 ng/g lipids	–	De Felip et al. (56)
	2007–2008	32	28–42	USA	0 (0.01) ng/mL	No association with IVF outcomes	Bloom et al. (60)
	2008–2009	40	25–43	Belgium	13 (3) pg/mL	No association with IVF outcomes	Petro et al. (57)
PCB 187	2000	12	28–32	Italy	63 ng/g lipids	–	De Felip et al. (56)
	2007–2008	32	28–42	USA	0.02 (0.02) ng/mL	No association with IVF outcomes	Bloom et al. (60)
	2008–2009	40	25–43	Belgium	18 (10) pg/mL	No association with IVF outcomes	Petro et al. (57)
PCB 189	2000	12	28–32	Italy	90 ng/g lipids	–	De Felip et al. (56)
PCB 194	2000	12	28–32	Italy	23 ng/g lipids	–	De Felip et al. (56)
PBDE 28	1994–2003	65	27–44	US	0.001 ng/g wet weight	No association with failed implantation	Johnson et al. (45)
	2013	127	20–35	China	ND	–	Huang et al. (63)
PBDE 47	1994–2003	65	27–44	US	0.026 ng/g wet weight	No association with failed implantation	Johnson et al. (45)
	2008–2009	40	25–43	Belgium	12 pg/mL	No association with IVF outcomes	Petro et al. (57)
	2013	127	20–35	China	0.32 (0.91) ng/g lipids	–	Huang et al. (63)
PBDE 99	1994–2003	65	27–44	US	0.014 ng/g wet weight	No association with failed implantation	Johnson et al. (45)
	2008–2009	40	25–43	Belgium	14 pg/mL	No association with IVF outcomes	Petro et al. (57)
	2013	127	20–35	China	13 (13) ng/g lipids	–	Huang et al. (63)
PBDE 100	1994–2003	65	27–44	US	0.006 ng/g wet weight	No association with failed implantation	Johnson et al. (45)
	2013	127	20–35	China	35 (21) ng/g lipids	–	Huang et al. (63)
PBDE 153	1994–2003	65	27–44	US	0.007 ng/g wet weight	Increased odds for failed implantation	Johnson et al. (45)
	2013	127	20–35	China	1.1 (3.3) ng/g lipids	–	Huang et al. (63)
PBDE 154	1994–2003	65	27–44	US	0.003 ng/g wet weight	No association with failed implantation	Johnson et al. (45)
	2013	127	20–35	China	0.43 (2.1) ng/g lipids	–	Huang et al. (63)
PBDE 183	1994–2003	65	27–44	US	0.000 ng/g wet weight	No association with failed implantation	Johnson et al. (45)
	2013	127	20–35	China	0.24 (1.1) ng/g lipids	–	Huang et al. (63)
PFOS	2008–2009	38	25–43	Belgium	[7.5] (30.3) ng/mL	Higher fertilization rate; higher proportion of high-quality embryo	Petro et al. (65)
	2015	59	20–45	UK	{2} (3.69) ng/mL	Irregular menstrual cycles; lower free androstenedione index	Heffernan et al. (66)
PFOA	2008–2009	38	25–43	Belgium	[1.8] (3) ng/mL	Higher fertilization rate; higher proportion of high-quality embryo	Petro et al. (66)
	2015	59	20–45	UK	{1.82} (6.21) ng/mL	Higher testosterone levels	Heffernan et al. (66)
PFNA	2008–2009	38	25–43	Belgium	[0.4] (1.9) ng/mL	Higher fertilization rate; higher proportion of high-quality embryo	Petro et al. (65)
	2015	59	20–45	UK	{0.41} (1.42) ng/mL	Higher testosterone and androstenedione levels	Heffernan et al. (66)
PFHxS	2008–2009	38	25–43	Belgium	[0.3] (1.3) ng/mL	Higher fertilization rate; higher proportion of high-quality embryos	Petro et al. (65)
	2015	59	20–45	UK	{0.88} (10) ng/mL	Higher testosterone levels	Heffernan et al. (66)

^a^Units used as reported in the study.

^b^An exposure study is indicated by –.

DDE: dichlorodiphenyldichloroethylene; DDT: dichlorodiphenyltrichloroethane; HCB: hexachlorobenzene; HCH: hexachlorocyclohexane; NR: not reported; PBDE: polybrominated diphenyl ether; PCB: polychlorinated biphenyl; POPs: persistent organic pollutants.

As these cohorts were composed of women undergoing IVF treatment, information on ovarian reserve, endometrial thickness, oocyte quality, fertilization rate, embryo quality, and live birth were readily available to further investigate the impact of POPs on human reproduction, specifically on IVF endpoints. Approximately half of the studies analysed association between exposure and outcome. Common outcomes evaluated were oocyte quality, implantation rate, and live birth as well as endometrial thickness. While some studies did not find any association between chemicals and IVF outcomes, others found that in particular the lipophilic POPs were associated with lower fertilisation rates and poorer embryo quality after adjusting for covariates such as age, body mass index, and oestradiol. For example, dichlorodiphenyldichloroethylene (DDE), a metabolite of DDT, was found to be associated with lower oocyte quality in three studies (43,57,60), while two failed to find associations (56,59). The indicator PCBs (PCBs 28, 52, 101, 138, 153, and 180) were associated with lower oestradiol, thinner endometrium, and lower fertilization rates in most studies (57,60,62). The PFAS compounds were evaluated in two studies and found to be associated with higher androgen levels and higher embryo quality (65,66). It is clear that more studies are warranted, both experimental and epidemiological, to interpret these associations. It should also be noted that the reported cohort studies are relatively small, most having fewer than 100 participants, which clearly limits the statistical power.

### 3.3. A way forward

Studying the effects of POPs on fertility in women is challenging, as fertility and fecundability depend on multiple factors. In addition, women (and couples) are exposed to multiple POPs, which makes statistical analyses challenging. Ideally, similar chemicals are grouped together, allowing comparison of toxic equivalency values which is currently done for dioxin and dioxin-like compounds. Alternatively, statistical methods that can handle highly correlated exposures and non-linear relationships that are typical for these chemicals should be further developed. Effects seen only in some quantiles of exposure should not be disregarded but rather explored further. Lastly, human folliculogenesis lasts for months, and during this time cytoplasmic and nuclear maturation take place including epigenetic changes and germline imprinting. Exposure assessment during preconception could help identify chemicals with adverse effects on oocyte quality.

Population studies give a good starting point for gauging associations between exposures and reproductive outcomes. However, for proving causality, experimental models will be needed. Better understanding of mechanisms underlying folliculogenesis, oocyte quality, ovarian aging, and endometrial receptivity will be needed in order to tailor better assays for chemical safety testing.

## 4. Conclusions

Multiple studies have identified cocktails of POPs in ovarian follicular fluid of reproductive-aged women across the world, although the use of most of the chemicals in focus in this review was restricted decades ago (Tables 1 and 2). Specifically, lipophilic organochlorine chemicals such as DDE and PCBs were associated with worse outcomes in IVF treatments (Table 2). In addition, these compounds have also been linked to other adverse reproductive outcomes in women (Table 1). Originally, organochlorine chemicals were found to be toxic for reproduction in wildlife animal populations. Our review suggests that they may also worsen the chances of successful fertility treatments in humans. Although cohort studies give information about significant associations between exposures and outcomes, they cannot demonstrate causality or inform about associated mechanisms. Therefore, experimental models will be needed for proving endocrine mechanisms of action as well as causality.

Because EDCs affect not only reproductive function of women but also the health of the offspring, it is of utmost importance that all action should be taken to reduce exposure. Advising women and families on the use of consumer products, healthy diets, and home supplies represents a good start and raises awareness. However, populations can only truly be protected with better chemical regulations that prevent harmful chemicals from entering the market. Therefore, research proving causal effects between EDC exposures and adverse effects in humans is urgently needed.

## Footnotes

1 A new estimate of chemicals in global commerce was published after this review was accepted, suggesting that there are 330,000 chemicals that are or have been in the market (3).
